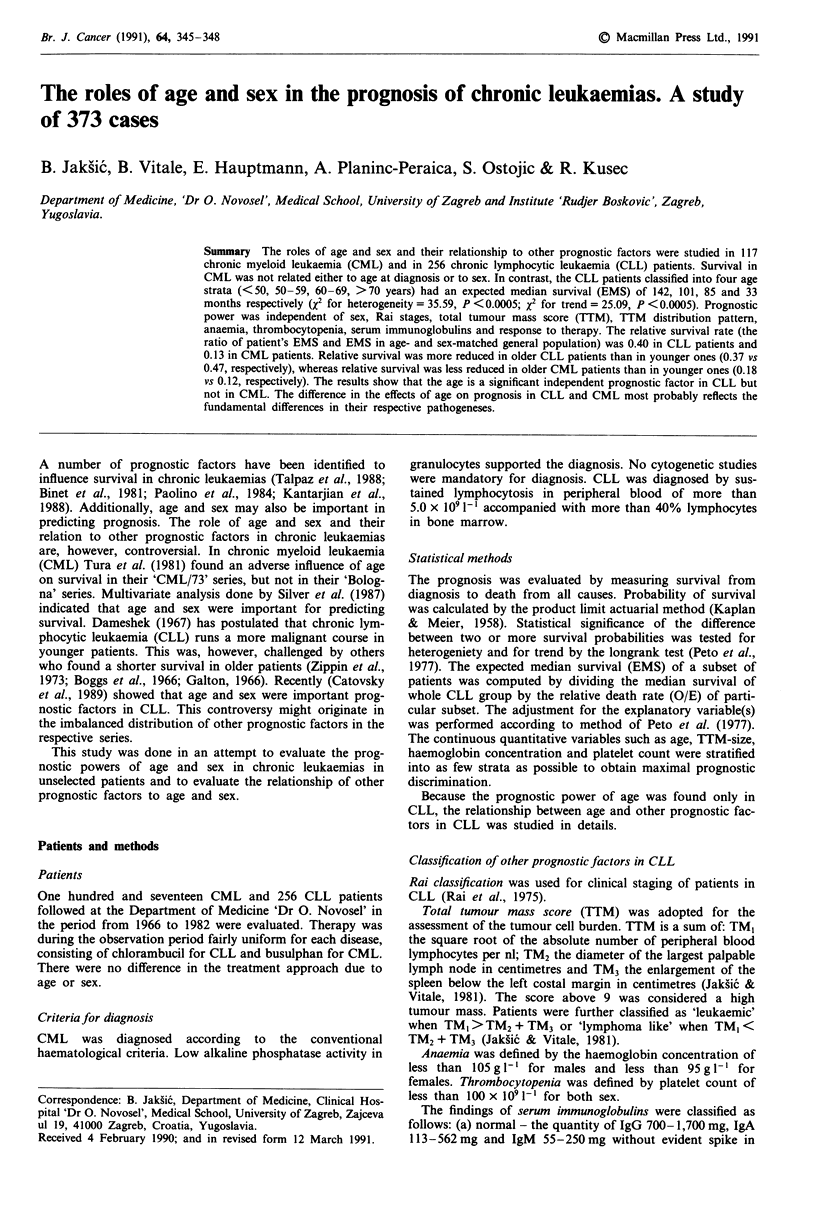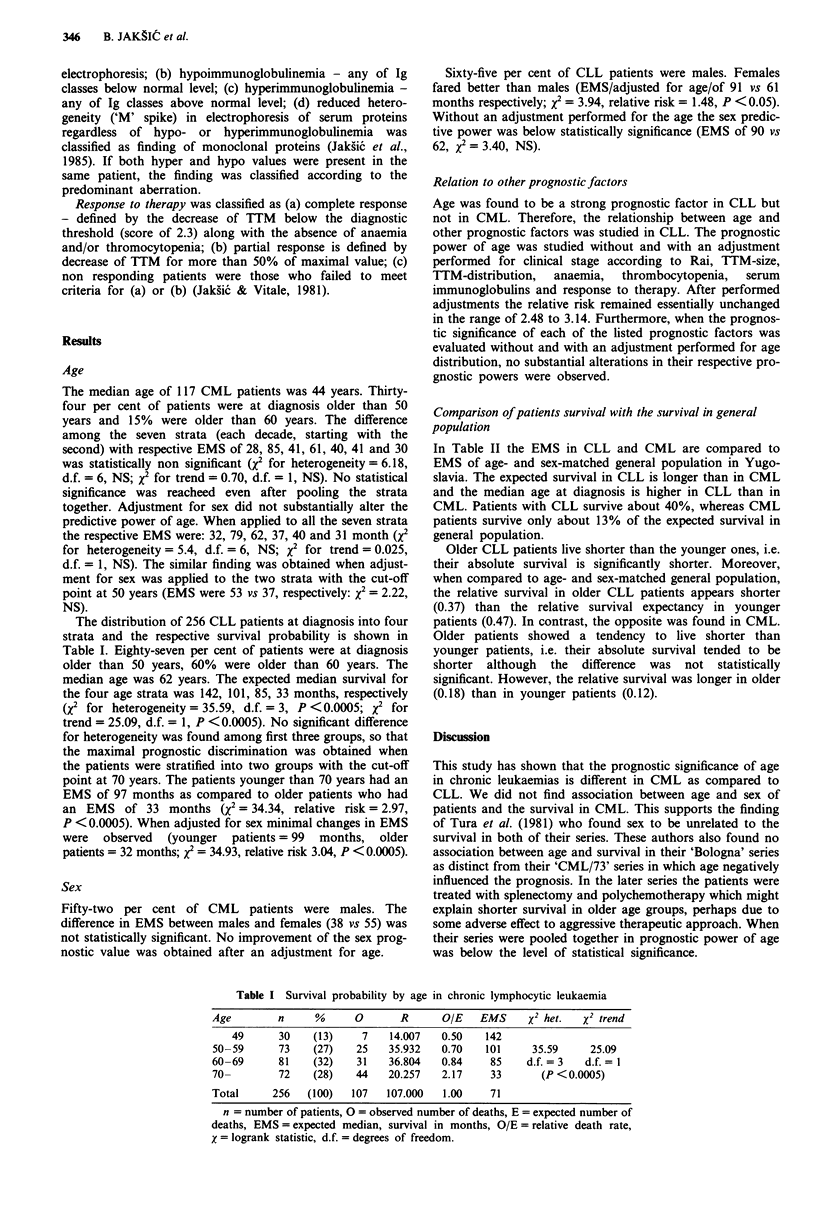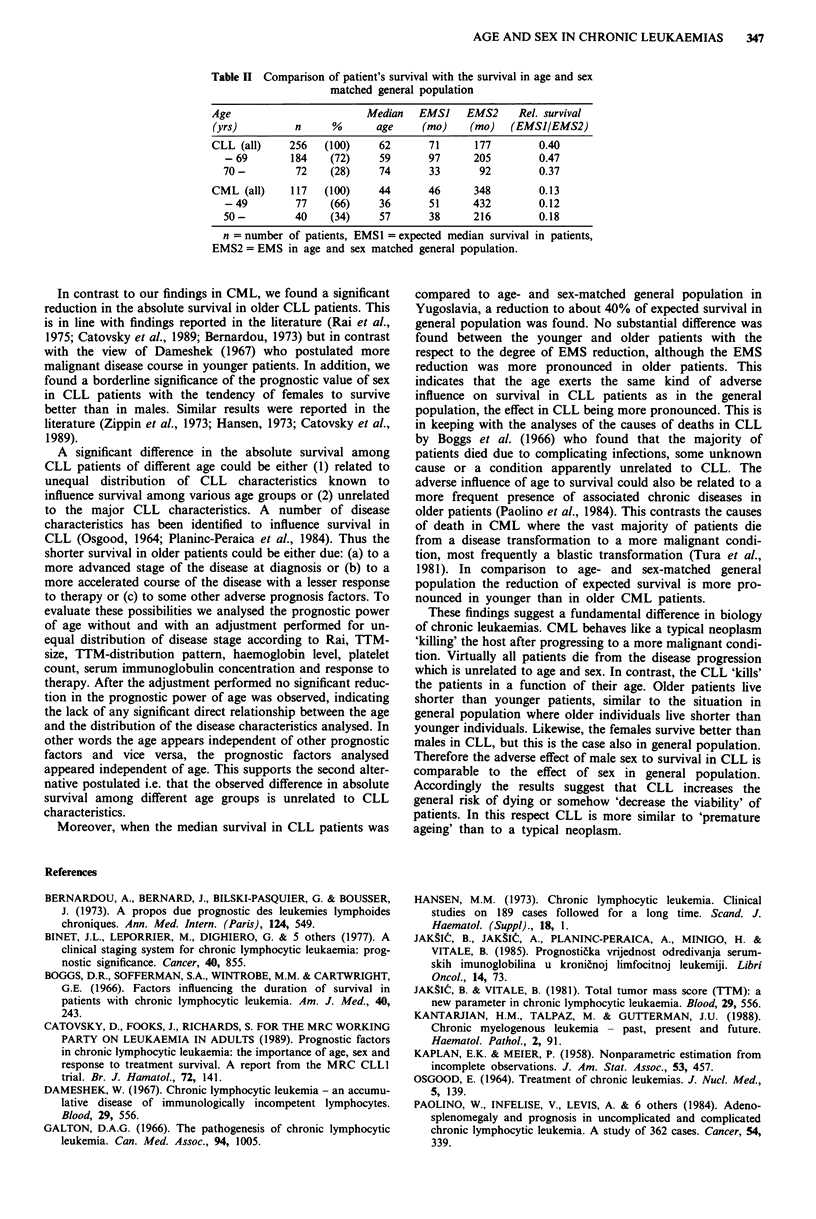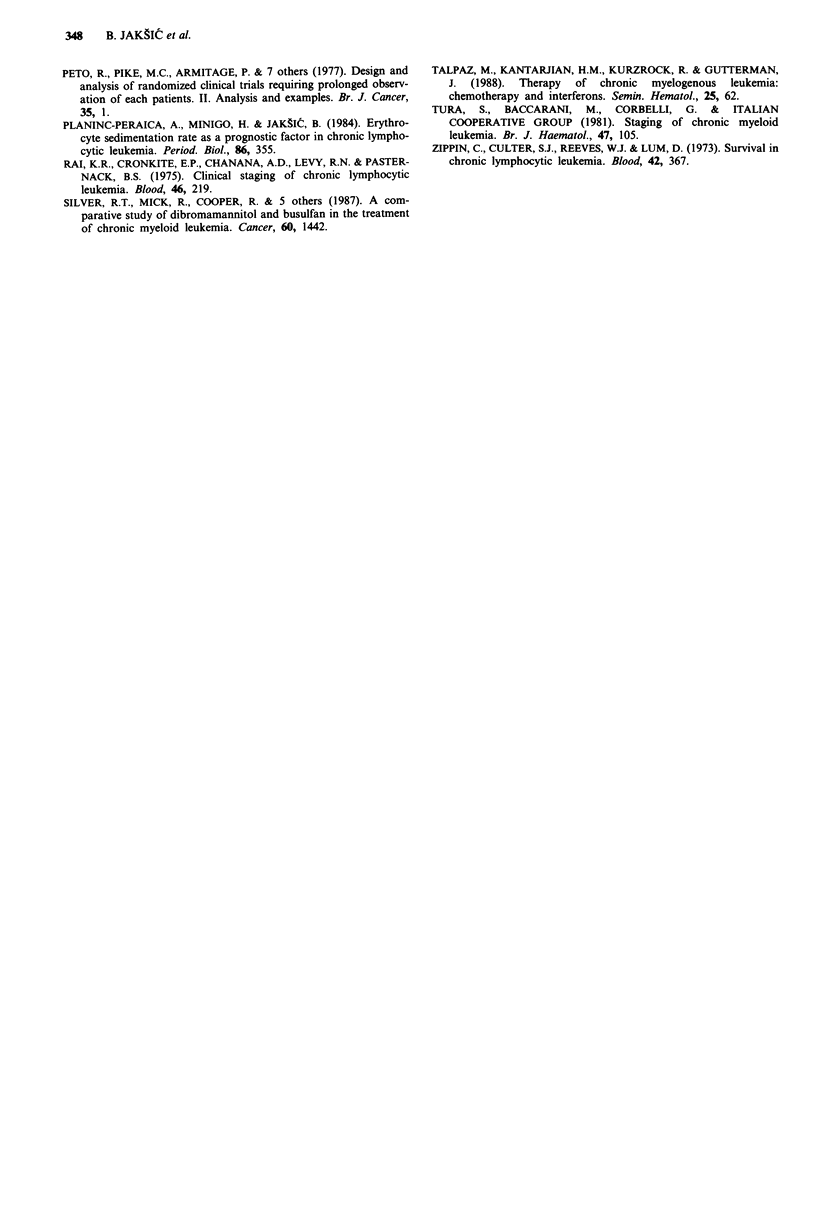# The roles of age and sex in the prognosis of chronic leukaemias. A study of 373 cases.

**DOI:** 10.1038/bjc.1991.303

**Published:** 1991-08

**Authors:** B. Jaksić, B. Vitale, E. Hauptmann, A. Planinc-Peraica, S. Ostojic, R. Kusec

**Affiliations:** Department of Medicine, Dr O. Novosel Medical School, University of Zagreb, Yugoslavia.

## Abstract

The roles of age and sex and their relationship to other prognostic factors were studied in 117 chronic myeloid leukaemia (CML) and in 256 chronic lymphocytic leukaemia (CLL) patients. Survival in CML was not related either to age at diagnosis or to sex. In contrast, the CLL patients classified into four age strata (less than 50, 50-59, 60-69, greater than 70 years) had an expected median survival (EMS) of 142, 101, 85 and 33 months respectively (chi 2 for heterogeneity = 35.59, P less than 0.0005; chi 2 for trend = 25.09, P less than 0.0005). Prognostic power was independent of sex, Rai stages, total tumour mass score (TTM), TTM distribution pattern, anaemia, thrombocytopenia, serum immunoglobulins and response to therapy. The relative survival rate (the ratio of patient's EMS and EMS in age- and sex-matched general population) was 0.40 in CLL patients and 0.13 in CML patients. Relative survival was more reduced in older CLL patients than in younger ones (0.37 vs 0.47, respectively), whereas relative survival was less reduced in older CML patients than in younger ones (0.18 vs 0.12, respectively). The results show that the age is a significant independent prognostic factor in CLL but not in CML. The difference in the effects of age on prognosis in CLL and CML most probably reflects the fundamental differences in their respective pathogeneses.


*Br. J. Cancer* (1991), **64**, 345–348 © Macmillan Press Ltd., 1991

### The roles of age and sex in the prognosis of chronic leukaemias. A study of 373 cases

B. Jakšić, B. Vitale, E. Hauptmann, A. Planinc-Peraica, S. Ostojic & R. Kusec

*Department of Medicine, 'Dr O. Novosel', Medical School, University of Zagreb and Institute 'Rudjer Boskovic', Zagreb, Yugoslavia.*

**Summary** The roles of age and sex and their relationship to other prognostic factors were studied in 117 chronic myeloid leukaemia (CML) and in 256 chronic lymphocytic leukaemia (CLL) patients. Survival in CML was not related either to age at diagnosis or to sex. In contrast, the CLL patients classified into four age strata (<50, 50–59, 60–69, >70 years) had an expected median survival (EMS) of 142, 101, 85 and 33 months respectively ($\chi^2$ for heterogeneity = 35.59, $P < 0.0005$; $\chi^2$ for trend = 25.09, $P < 0.0005$). Prognostic power was independent of sex, Rai stages, total tumour mass score (TTM), TTM distribution pattern, anaemia, thrombocytopenia, serum immunoglobulins and response to therapy. The relative survival rate (the ratio of patient's EMS and EMS in age- and sex-matched general population) was 0.40 in CLL patients and 0.13 in CML patients. Relative survival was more reduced in older CLL patients than in younger ones (0.37 *vs* 0.47, respectively), whereas relative survival was less reduced in older CML patients than in younger ones (0.18 *vs* 0.12, respectively). The results show that the age is a significant independent prognostic factor in CLL but not in CML. The difference in the effects of age on prognosis in CLL and CML most probably reflects the fundamental differences in their respective pathogeneses.

A number of prognostic factors have been identified to influence survival in chronic leukaemias (Talpaz *et al.*, 1988; Binet *et al.*, 1981; Paolino *et al.*, 1984; Kantarjian *et al.*, 1988). Additionally, age and sex may also be important in predicting prognosis. The role of age and sex and their relation to other prognostic factors in chronic leukaemias are, however, controversial. In chronic myeloid leukaemia (CML) Tura *et al.* (1981) found an adverse influence of age on survival in their 'CML/73' series, but not in their 'Bologna' series. Multivariate analysis done by Silver *et al.* (1987) indicated that age and sex were important for predicting survival. Dameshek (1967) has postulated that chronic lymphocytic leukaemia (CLL) runs a more malignant course in younger patients. This was, however, challenged by others who found a shorter survival in older patients (Zippin *et al.*, 1973; Boggs *et al.*, 1966; Galton, 1966). Recently (Catovsky *et al.*, 1989) showed that age and sex were important prognostic factors in CLL. This controversy might originate in the imbalanced distribution of other prognostic factors in the respective series.

This study was done in an attempt to evaluate the prognostic powers of age and sex in chronic leukaemias in unselected patients and to evaluate the relationship of other prognostic factors to age and sex.

### Patients and methods

### *Patients*

One hundred and seventeen CML and 256 CLL patients followed at the Department of Medicine 'Dr O. Novosel' in the period from 1966 to 1982 were evaluated. Therapy was during the observation period fairly uniform for each disease, consisting of chlorambucil for CLL and busulphan for CML. There were no difference in the treatment approach due to age or sex.

### *Criteria for diagnosis*

CML was diagnosed according to the conventional haematological criteria. Low alkaline phosphatase activity in granulocytes supported the diagnosis. No cytogenetic studies were mandatory for diagnosis. CLL was diagnosed by sustained lymphocytosis in peripheral blood of more than $5.0 \times 10^9\,l^{-1}$ accompanied with more than 40% lymphocytes in bone marrow.

### *Statistical methods*

The prognosis was evaluated by measuring survival from diagnosis to death from all causes. Probability of survival was calculated by the product limit actuarial method (Kaplan & Meier, 1958). Statistical significance of the difference between two or more survival probabilities was tested for heterogeniety and for trend by the longrank test (Peto *et al.*, 1977). The expected median survival (EMS) of a subset of patients was computed by dividing the median survival of whole CLL group by the relative death rate (O/E) of particular subset. The adjustment for the explanatory variable(s) was performed according to method of Peto *et al.* (1977). The continuous quantitative variables such as age, TTM-size, haemoglobin concentration and platelet count were stratified into as few strata as possible to obtain maximal prognostic discrimination.

Because the prognostic power of age was found only in CLL, the relationship between age and other prognostic factors in CLL was studied in details.

### *Classification of other prognostic factors in CLL*

*Rai classification* was used for clinical staging of patients in CLL (Rai *et al.*, 1975).

*Total tumour mass score* (TTM) was adopted for the assessment of the tumour cell burden. TTM is a sum of: $TM_1$ the square root of the absolute number of peripheral blood lymphocytes per nl; $TM_2$ the diameter of the largest palpable lymph node in centimetres and $TM_3$ the enlargement of the spleen below the left costal margin in centimetres (Jakšić & Vitale, 1981). The score above 9 was considered a high tumour mass. Patients were further classified as 'leukaemic' when $TM_1 > TM_2 + TM_3$ or 'lymphoma like' when $TM_1 < TM_2 + TM_3$ (Jakšić & Vitale, 1981).

*Anaemia* was defined by the haemoglobin concentration of less than $105\,g\,l^{-1}$ for males and less than $95\,g\,l^{-1}$ for females. *Thrombocytopenia* was defined by platelet count of less than $100 \times 10^9\,l^{-1}$ for both sex.

The findings of *serum immunoglobulins* were classified as follows: (a) normal – the quantity of IgG 700–1,700 mg, IgA 113–562 mg and IgM 55–250 mg without evident spike in

Correspondence: B. Jakšić, Department of Medicine, Clinical Hospital 'Dr O. Novosel', Medical School, University of Zagreb, Zajceva ul 19, 41000 Zagreb, Croatia, Yugoslavia.
Received 4 February 1990; and in revised form 12 March 1991.

electrophoresis; (b) hypoimmunoglobulinemia – any of Ig classes below normal level; (c) hyperimmunoglobulinemia – any of Ig classes above normal level; (d) reduced heterogeneity ('M' spike) in electrophoresis of serum proteins regardless of hypo- or hyperimmunoglobulinemia was classified as finding of monoclonal proteins (Jakšić *et al.*, 1985). If both hyper and hypo values were present in the same patient, the finding was classified according to the predominant aberration.

*Response to therapy* was classified as (a) complete response – defined by the decrease of TTM below the diagnostic threshold (score of 2.3) along with the absence of anaemia and/or thromocytopenia; (b) partial response is defined by decrease of TTM for more than 50% of maximal value; (c) non responding patients were those who failed to meet criteria for (a) or (b) (Jakšić & Vitale, 1981).

### Results

### *Age*

The median age of 117 CML patients was 44 years. Thirty-four per cent of patients were at diagnosis older than 50 years and 15% were older than 60 years. The difference among the seven strata (each decade, starting with the second) with respective EMS of 28, 85, 41, 61, 40, 41 and 30 was statistically non significant ($\chi^2$ for heterogeneity = 6.18, d.f. = 6, NS; $\chi^2$ for trend = 0.70, d.f. = 1, NS). No statistical significance was reacheed even after pooling the strata together. Adjustment for sex did not substantially alter the predictive power of age. When applied to all the seven strata the respective EMS were: 32, 79, 62, 37, 40 and 31 month ($\chi^2$ for heterogeneity = 5.4, d.f. = 6, NS; $\chi^2$ for trend = 0.025, d.f. = 1, NS). The similar finding was obtained when adjustment for sex was applied to the two strata with the cut-off point at 50 years (EMS were 53 *vs* 37, respectively: $\chi^2$ = 2.22, NS).

The distribution of 256 CLL patients at diagnosis into four strata and the respective survival probability is shown in Table I. Eighty-seven per cent of patients were at diagnosis older than 50 years, 60% were older than 60 years. The median age was 62 years. The expected median survival for the four age strata was 142, 101, 85, 33 months, respectively ($\chi^2$ for heterogeneity = 35.59, d.f. = 3, $P < 0.0005$; $\chi^2$ for trend = 25.09, d.f. = 1, $P < 0.0005$). No significant difference for heterogeneity was found among first three groups, so that the maximal prognostic discrimination was obtained when the patients were stratified into two groups with the cut-off point at 70 years. The patients younger than 70 years had an EMS of 97 months as compared to older patients who had an EMS of 33 months ($\chi^2$ = 34.34, relative risk = 2.97, $P < 0.0005$). When adjusted for sex minimal changes in EMS were observed (younger patients = 99 months, older patients = 32 months; $\chi^2$ = 34.93, relative risk 3.04, $P < 0.0005$).

### *Sex*

Fifty-two per cent of CML patients were males. The difference in EMS between males and females (38 *vs* 55) was not statistically significant. No improvement of the sex prognostic value was obtained after an adjustment for age.

Sixty-five per cent of CLL patients were males. Females fared better than males (EMS/adjusted for age/of 91 *vs* 61 months respectively; $\chi^2$ = 3.94, relative risk = 1.48, $P < 0.05$). Without an adjustment performed for the age the sex predictive power was below statistically significance (EMS of 90 *vs* 62, $\chi^2$ = 3.40, NS).

### *Relation to other prognostic factors*

Age was found to be a strong prognostic factor in CLL but not in CML. Therefore, the relationship between age and other prognostic factors was studied in CLL. The prognostic power of age was studied without and with an adjustment performed for clinical stage according to Rai, TTM-size, TTM-distribution, anaemia, thrombocytopenia, serum immunoglobulins and response to therapy. After performed adjustments the relative risk remained essentially unchanged in the range of 2.48 to 3.14. Furthermore, when the prognostic significance of each of the listed prognostic factors was evaluated without and with an adjustment performed for age distribution, no substantial alterations in their respective prognostic powers were observed.

### *Comparison of patients survival with the survival in general population*

In Table II the EMS in CLL and CML are compared to EMS of age- and sex-matched general population in Yugoslavia. The expected survival in CLL is longer than in CML and the median age at diagnosis is higher in CLL than in CML. Patients with CLL survive about 40%, whereas CML patients survive only about 13% of the expected survival in general population.

Older CLL patients live shorter than the younger ones, i.e. their absolute survival is significantly shorter. Moreover, when compared to age- and sex-matched general population, the relative survival in older CLL patients appears shorter (0.37) than the relative survival expectancy in younger patients (0.47). In contrast, the opposite was found in CML. Older patients showed a tendency to live shorter than younger patients, i.e. their absolute survival tended to be shorter although the difference was not statistically significant. However, the relative survival was longer in older (0.18) than in younger patients (0.12).

### Discussion

This study has shown that the prognostic significance of age in chronic leukaemias is different in CML as compared to CLL. We did not find association between age and sex of patients and the survival in CML. This supports the finding of Tura *et al.* (1981) who found sex to be unrelated to the survival in both of their series. These authors also found no association between age and survival in their 'Bologna' series as distinct from their 'CML/73' series in which age negatively influenced the prognosis. In the later series the patients were treated with splenectomy and polychemotherapy which might explain shorter survival in older age groups, perhaps due to some adverse effect to aggressive therapeutic approach. When their series were pooled together in prognostic power of age was below the level of statistical significance.

**Table I** Survival probability by age in chronic lymphocytic leukaemia

| *Age* | *n* | *%* | *O* | *R* | *O/E* | *EMS* | $\chi^2$ *het.* | $\chi^2$ *trend* |
|---|---|---|---|---|---|---|---|---|
| 49 | 30 | (13) | 7 | 14.007 | 0.50 | 142 | | |
| 50–59 | 73 | (27) | 25 | 35.932 | 0.70 | 101 | 35.59 | 25.09 |
| 60–69 | 81 | (32) | 31 | 36.804 | 0.84 | 85 | d.f. = 3 | d.f. = 1 |
| 70– | 72 | (28) | 44 | 20.257 | 2.17 | 33 | ($P < 0.0005$) | |
| Total | 256 | (100) | 107 | 107.000 | 1.00 | 71 | | |

*n* = number of patients, O = observed number of deaths, E = expected number of deaths, EMS = expected median, survival in months, O/E = relative death rate, $\chi$ = logrank statistic, d.f. = degrees of freedom.

**Table II** Comparison of patient's survival with the survival in age and sex matched general population

| *Age (yrs)* | *n* | *%* | *Median age* | *EMS1 (mo)* | *EMS2 (mo)* | *Rel. survival (EMS1/EMS2)* |
|---|---|---|---|---|---|---|
| CLL (all) | 256 | (100) | 62 | 71 | 177 | 0.40 |
| – 69 | 184 | (72) | 59 | 97 | 205 | 0.47 |
| 70 – | 72 | (28) | 74 | 33 | 92 | 0.37 |
| CML (all) | 117 | (100) | 44 | 46 | 348 | 0.13 |
| – 49 | 77 | (66) | 36 | 51 | 432 | 0.12 |
| 50 – | 40 | (34) | 57 | 38 | 216 | 0.18 |

*n* = number of patients, EMS1 = expected median survival in patients, EMS2 = EMS in age and sex matched general population.

In contrast to our findings in CML, we found a significant reduction in the absolute survival in older CLL patients. This is in line with findings reported in the literature (Rai *et al.*, 1975; Catovsky *et al.*, 1989; Bernardou, 1973) but in contrast with the view of Dameshek (1967) who postulated more malignant disease course in younger patients. In addition, we found a borderline significance of the prognostic value of sex in CLL patients with the tendency of females to survive better than in males. Similar results were reported in the literature (Zippin *et al.*, 1973; Hansen, 1973; Catovsky *et al.*, 1989).

A significant difference in the absolute survival among CLL patients of different age could be either (1) related to unequal distribution of CLL characteristics known to influence survival among various age groups or (2) unrelated to the major CLL characteristics. A number of disease characteristics has been identified to influence survival in CLL (Osgood, 1964; Planinc-Peraica *et al.*, 1984). Thus the shorter survival in older patients could be either due: (a) to a more advanced stage of the disease at diagnosis or (b) to a more accelerated course of the disease with a lesser response to therapy or (c) to some other adverse prognosis factors. To evaluate these possibilities we analysed the prognostic power of age without and with an adjustment performed for unequal distribution of disease stage according to Rai, TTM-size, TTM-distribution pattern, haemoglobin level, platelet count, serum immunoglobulin concentration and response to therapy. After the adjustment performed no significant reduction in the prognostic power of age was observed, indicating the lack of any significant direct relationship between the age and the distribution of the disease characteristics analysed. In other words the age appears independent of other prognostic factors and vice versa, the prognostic factors analysed appeared independent of age. This supports the second alternative postulated i.e. that the observed difference in absolute survival among different age groups is unrelated to CLL characteristics.

Moreover, when the median survival in CLL patients was compared to age- and sex-matched general population in Yugoslavia, a reduction to about 40% of expected survival in general population was found. No substantial difference was found between the younger and older patients with the respect to the degree of EMS reduction, although the EMS reduction was more pronounced in older patients. This indicates that the age exerts the same kind of adverse influence on survival in CLL patients as in the general population, the effect in CLL being more pronounced. This is in keeping with the analyses of the causes of deaths in CLL by Boggs *et al.* (1966) who found that the majority of patients died due to complicating infections, some unknown cause or a condition apparently unrelated to CLL. The adverse influence of age to survival could also be related to a more frequent presence of associated chronic diseases in older patients (Paolino *et al.*, 1984). This contrasts the causes of death in CML where the vast majority of patients die from a disease transformation to a more malignant condition, most frequently a blastic transformation (Tura *et al.*, 1981). In comparison to age- and sex-matched general population the reduction of expected survival is more pronounced in younger than in older CML patients.

These findings suggest a fundamental difference in biology of chronic leukaemias. CML behaves like a typical neoplasm 'killing' the host after progressing to a more malignant condition. Virtually all patients die from the disease progression which is unrelated to age and sex. In contrast, the CLL 'kills' the patients in a function of their age. Older patients live shorter than younger patients, similar to the situation in general population where older individuals live shorter than younger individuals. Likewise, the females survive better than males in CLL, but this is the case also in general population. Therefore the adverse effect of male sex to survival in CLL is comparable to the effect of sex in general population. Accordingly the results suggest that CLL increases the general risk of dying or somehow 'decrease the viability' of patients. In this respect CLL is more similar to 'premature ageing' than to a typical neoplasm.

### References

BERNARDOU, A., BERNARD, J., BILSKI-PASQUIER, G. & BOUSSER, J. (1973). A propos due prognostic des leukemies lymphoides chroniques. *Ann. Med. Intern. (Paris)*, **124**, 549.

BINET, J.L., LEPORRIER, M., DIGHIERO, G. & 5 others (1977). A clinical staging system for chronic lymphocytic leukaemia: prognostic significance. *Cancer*, **40**, 855.

BOGGS, D.R., SOFFERMAN, S.A., WINTROBE, M.M. & CARTWRIGHT, G.E. (1966). Factors influencing the duration of survival in patients with chronic lymphocytic leukemia. *Am. J. Med.*, **40**, 243.

CATOVSKY, D., FOOKS, J., RICHARDS, S. FOR THE MRC WORKING PARTY ON LEUKAEMIA IN ADULTS (1989). Prognostic factors in chronic lymphocytic leukaemia: the importance of age, sex and response to treatment survival. A report from the MRC CLL1 trial. *Br. J. Hamatol.*, **72**, 141.

DAMESHEK, W. (1967). Chronic lymphocytic leukemia – an accumulative disease of immunologically incompetent lymphocytes. *Blood*, **29**, 556.

GALTON, D.A.G. (1966). The pathogenesis of chronic lymphocytic leukemia. *Can. Med. Assoc.*, **94**, 1005.

HANSEN, M.M. (1973). Chronic lymphocytic leukemia. Clinical studies on 189 cases followed for a long time. *Scand. J. Haematol. (Suppl)*., **18**, 1.

JAKŠIĆ, B., JAKŠIĆ, A., PLANINC-PERAICA, A., MINIGO, H. & VITALE, B. (1985). Prognostička vrijednost odredivanja serumskih imunoglobilina u kroničnoj limfocitnoj leukemiji. *Libri Oncol.*, **14**, 73.

JAKŠIĆ, B. & VITALE, B. (1981). Total tumor mass score (TTM): a new parameter in chronic lymphocytic leukaemia. *Blood*, **29**, 556.

KANTARJIAN, H.M., TALPAZ, M. & GUTTERMAN, J.U. (1988). Chronic myelogenous leukemia – past, present and future. *Haematol. Pathol.*, **2**, 91.

KAPLAN, E.K. & MEIER, P. (1958). Nonparametric estimation from incomplete observations. *J. Am. Stat. Assoc.*, **53**, 457.

OSGOOD, E. (1964). Treatment of chronic leukemias. *J. Nucl. Med.*, **5**, 139.

PAOLINO, W., INFELISE, V., LEVIS, A. & 6 others (1984). Adenosplenomegaly and prognosis in uncomplicated and complicated chronic lymphocytic leukemia. A study of 362 cases. *Cancer*, **54**, 339.

PETO, R., PIKE, M.C., ARMITAGE, P. & 7 others (1977). Design and analysis of randomized clinical trials requiring prolonged observation of each patients. II. Analysis and examples. *Br. J. Cancer*, **35**, 1.

PLANINC-PERAICA, A., MINIGO, H. & JAKŠIĆ, B. (1984). Erythrocyte sedimentation rate as a prognostic factor in chronic lymphocytic leukemia. *Period. Biol.*, **86**, 355.

RAI, K.R., CRONKITE, E.P., CHANANA, A.D., LEVY, R.N. & PASTERNACK, B.S. (1975). Clinical staging of chronic lymphocytic leukemia. *Blood*, **46**, 219.

SILVER, R.T., MICK, R., COOPER, R. & 5 others (1987). A comparative study of dibromamannitol and busulfan in the treatment of chronic myeloid leukemia. *Cancer*, **60**, 1442.

TALPAZ, M., KANTARJIAN, H.M., KURZROCK, R. & GUTTERMAN, J. (1988). Therapy of chronic myelogenous leukemia: chemotherapy and interferons. *Semin. Hematol.*, **25**, 62.

TURA, S., BACCARANI, M., CORBELLI, G. & ITALIAN COOPERATIVE GROUP (1981). Staging of chronic myeloid leukemia. *Br. J. Haematol.*, **47**, 105.

ZIPPIN, C., CULTER, S.J., REEVES, W.J. & LUM, D. (1973). Survival in chronic lymphocytic leukemia. *Blood*, **42**, 367.